# The Effect of Work Characteristics on Dermatologic Symptoms in Hairdressers

**DOI:** 10.1186/2052-4374-26-13

**Published:** 2014-06-09

**Authors:** Pil Kyun Jung, June-Hee Lee, Ji Hye Baek, Jungho Hwang, Jong-Uk Won, Inah Kim, Jaehoon Roh

**Affiliations:** 1 Graduate School of Public Health, Yonsei University, Seoul, Korea; 2 The Institute for Occupational Health, Yonsei University College of Medicine, Seoul, Korea; 3 Korea Occupational Safety and Health Agency (KOSHA), Service Industry Safety Team, Chungbuk Area Office, Ulsan, Korea; 4 Department of Preventive Medicine and Public Health, Yonsei University College of Medicine, Seoul, Korea

**Keywords:** Hair preparations, Occupational health, Dermatitis, occupational, Eczema

## Abstract

**Objectives:**

Hairdressers in Korea perform various tasks and are exposed to health risk factors such as chemical substances or prolonged duration of wet work. The objective of this study is to provide descriptive statistics on the demographics and work characteristics of hairdressers in Korea and to identify work-related risk factors for dermatologic symptoms in hairdressers.

**Methods:**

1,054 hairdressers were selected and analyzed for this study. Independent variables were exposure to chemical substances, the training status of the hairdressers, and the main tasks required of them, and the dependent variable was the incidence of dermatologic symptoms. The relationships between work characteristics and dermatologic symptoms were evaluated by estimating odds ratios using multiple logistic regression analysis.

**Results:**

Among the 1,054 study subjects, 212 hairdressers (20.1%) complained of dermatologic symptoms, and the symptoms were more prevalent in younger, unmarried or highly educated hairdressers. The main tasks that comprise the majority of the wet work were strictly determined by training status, since 96.5% of staff hairdressers identified washing as their main task, while only 1.5% and 2.0% of master and designer hairdressers, respectively, identified this as their main task. Multiple logistic regressions was performed to estimate odds ratios. While exposure to hairdressing chemicals showed no significant effect on the odds ratio for the incidence of dermatologic symptoms, higher odds ratios of dermatologic symptoms were shown in staff hairdressers (2.70, 95% CI: 1.32 - 5.51) and in hairdressers who perform washing as their main task (2.03, 95% CI: 1.22 - 3.37), after adjusting for general and work characteristics.

**Conclusions:**

This study showed that the training status and main tasks of hairdressers are closely related to each other and that the training status and main tasks of hairdressers are related to the incidence of dermatologic symptoms. This suggests that in the future, regulations on working conditions and health management guidelines for hairdressers should be established.

## Introduction

According to the nationwide Survey for Wholesale and Retail Trade/Service Industry by Statistics Korea in 2011, 126,358 hairdressers were working in 81,671 hair salons, and this figure has been increasing since 2006 [1]. Hairdressers are known to be exposed to over 3,000 kinds of chemicals, about 30% of which are classified as being toxic to humans [2]. Hairdressers also experience physical health risk factors such as unfavorable work postures; for example, working with arms raised, or remaining in a standing position for a prolonged time. In addition, hairdressing involves certain amounts of repetitive movement of the hands or arms [3,4]. In addition to these risk factors, hairdressers are also exposed to prolonged wet work [5] since a considerable portion of hairdressers’ tasks involves water-resistant glove wearing or direct contact with water [6]. There have been some previous studies regarding health issues in hairdressers in Korea, such as musculoskeletal disease [7], chemical exposure [8] or dermatologic disease [9].

Contact dermatitis is a multifactorial disease [10]. While irritants or a harsh working environment as mentioned above act as exogenous risk factors, innate immune reactivity or a history of atopic disease could act as endogenous risk factors [11]. Contact dermatitis is characterized by symptoms such as skin dryness, fissuring, itching, or hyperkeratosis, and these symptoms may last for several years, even if the irritants are removed [12]. Factors such as water, detergents, acids, alkalis, or cold friction can all contribute to the initiation of contact dermatitis [13,14], and these factors are commonly associated with wet work [15]. Individuals who are exposed to wet work for more than 2 hours per day, or who use water-resistant gloves, are considered to be exposed to wet work [16]. People in occupations that involve exposure to wet work include cleaners [17], health care workers [18] and hairdressers [19].

Reports of dermatitis in hairdressers in various cultures are not uncommon, and the results of previous studies show a cumulative prevalence of 17-42% [20,21]. Some studies suggest that dermatitis in hairdressers is common in groups with certain levels of training due to differences in the frequency of the main tasks in the job that involve wet work [22], but other studies show contradictory results concerning the training status and main tasks of the hairdressers [5].

Although this is a profound health issue in hairdressers, there are very few studies of the relationship between work characteristics and dermatitis in hairdressers in Korea. Thus, the aim of this study is to identify the relationships between individual work characteristics and the relationship between work characteristics and dermatologic symptoms in hairdressers in Korea.

## Materials and methods

### Study subjects

The survey was conducted for five months, from May to September in 2012, using self-administered questionnaires. About 1% of the hairdressers in Korea were randomly selected for this study, and the study population was selected proportionally according to business type, size of the hairdressing salon, and training status, in order to obtain a representative sample. A total of 1,500 questionnaires were distributed through relevant hairdresser associations and academic organizations, and 1,209 questionnaires were returned, giving a response rate of 80.6%. 1,054 questionnaires were used in the final analysis due to missing values in relevant sections (dermatologic symptoms, training status, or main task) of the questionnaire. All study procedures were approved by the Yonsei University Graduate School of Public Health Institutional Review Board (IRB 2013-A-025).

### Study variables and measurements

The questionnaire used in this research consisted of two sections: general characteristics and work characteristics. The questions regarding general characteristics included demographic variables and lifestyle-related risk factors. Demographic variables included age, gender, BMI, marital status, and educational level. Lifestyle-related risk factors included smoking status, weekly alcohol consumption, the amount of regular exercise and perceived state of health.

Regarding marital status, “not married” was defined as people who were either not married or had been married in the past but were no longer married, including people who were divorced or bereaved spouses. Regarding weekly alcohol consumption, “none” was defined as the consumption by people who do not drink or who only drink on rare occasions, “mild” was defined as the consumption by people who drink 4 times or less per week and “heavy” was defined as the consumption by those who drink 5 times or more per week. The perceived state of health of the participants was categorized into three groups: those who answered “very good” or “good” to the relevant question were categorized as “good”; those who answered “normal” to the relevant question were categorized as “Normal”; and the remainders were categorized as “bad”. The “main task” was defined as the most frequently performed work task. Work tenure was defined as the total duration of working as a hairdresser. Working hours were calculated in terms of a working week. Over-sleeves, aprons, or other types of miscellaneous personal protective equipment were included in the category of “other” when they were mentioned in the answer to the question about the type of equipment used.

Regarding the questions that were intended to evaluate the incidence of dermatologic symptoms in hairdressers in the past year, a total of 5 questions that represent the symptoms of contact dermatitis were adopted from previous research on the health hazards and occupational accidents of firefighters. The first four of these questions were as follows: 1) “Have you experienced redness and swelling?”; 2) “Have you experienced redness and cracking?”; 3) “Have you experienced blister formations?”; and 4) “Have you experienced redness and itching?”. Clinically, the symptoms of contact dermatitis are known to last for over 3 weeks and to persist after the removal of irritants;, thus participants who answered “yes” to any one of the above questions were asked to answer the following question: “Have you experienced any of these symptoms for more than three weeks?”. Participants who answered “yes” to this final question were categorized into a group that had experienced dermatologic symptoms.

### Statistical analysis

The general and work characteristics of the study subjects were evaluated and the differences in the incidence of dermatologic symptoms among the independent variables were assessed by the Student’s *t*-test. Correlation analysis using Pearson’s coefficients to evaluate relatedness between the variables which are assumed to be closely linked to each other, such as age and work tenure or business type and salon size, was performed, and odds ratios (OR) with a 95% confidence interval (95% CI) of dermatologic symptoms were estimated using multiple logistic regression analysis. Model I was adjusted for general characteristics such as age, gender, marital status, educational level, smoking status, weekly alcohol consumption, amount of regular exercise, and perceived state of health. Model II was additionally adjusted for work characteristics such as business type, exposure to hairdressing chemicals and whether personal protective equipment was used. Work tenure and salon size were not used to adjust the regression model, since these two variables showed high correlations with training status (Pearson’s coefficient: 0.74, p-value < 0.001) and business type (Pearson’s coefficient: 0.75, p-value < 0.001). The risks were expressed as odds ratios in relation to both the reference group of hairdressing masters and to the cutting work group. All analyses were two-tailed and p values less than 0.05 were regarded as statistically significant. All statistical tests were performed using SAS software, version 9.2 (SAS Institute, Cary, NC).

## Results

The mean age of the study subjects was 36.9 ± 10.4 years and the majority (85.6%) was female (894 hairdressers). Only 42 (4.4%) of the study subjects were obese (BMI > 25). 41.0% (410 hairdressers) were married, and 45.4% (471 hairdressers) were high school graduates. Regarding lifestyle-related risk factors, 21.0% (214 hairdressers) of the study subjects were current smokers, 7.8% (77 hairdressers) were heavy drinkers, 39.3% (401 hairdressers) performed regular exercise and 13.1% (136 hairdressers) perceived their state of health to be in bad condition. Regarding work characteristics, 25.6% (234 hairdressers) of hairdressers worked more than 52 hours per week and 19.8% (201 hairdressers) were exposed to chemicals during working hours. Regarding the training status of the study subjects, 388 hairdressers (36.8%) were masters, 380 (36.1%) were designers, and 286 (27.1%) were staff hairdressers (Table 1, Table 2).

**Table 1:** Demographic and health characteristics of study subjects

Variable^*^	**n**	**(%)**
Age		
< 30	342	(33.4)
< 40	334	(32.6)
≧ 40	349	(34.0)
Gender		
Male	151	(14.4)
Female	894	(85.6)
BMI^†^		
< 25	916	(95.6)
≧ 25	42	(4.4)
Marital status		
Not married^‡^	591	(59.0)
Married	410	(41.0)
Educational level		
≦ High school completion	566	(54.6)
> High school completion	471	(45.4)
Smoking status		
Non/past smoker	805	(79.0)
Current smoker	214	(21.0)
Weekly alcohol consumption^§^		
None	453	(45.8)
Mild	460	(46.5)
Heavy	77	(7.8)
Weekly exercise		
None	619	(60.7)
≦ 4times	345	(33.8)
> 4times	56	(5.5)
Perceived state of health		
Good	410	(39.6)
Normal	489	(47.2)
Bad	136	(13.1)

^*^The total of each variable is not always 1,054 due to missing values.

^†^Body mass index: weight/height^2^.

^‡^Includes divorced, separated and bereaved.

^§^None: never drink or only on rare occasions, Mild: < 4 times per week, Heavy: ≧ 5 times per week.

**Table 2:** Work characteristics of study subjects

Variable^*^	**n**	**(%)**
Business type		
Franchise hair salons	433	(42.2)
Private hair salons	594	(57.8)
Salon size (number of employees)		
1	228	(23.5)
2 - 4	248	(25.6)
5 - 9	146	(15.1)
≧ 10	347	(35.8)
Training status		
Master	388	(36.8)
Designer	380	(36.1)
Staff	286	(27.1)
Main task		
Cutting	620	(58.8)
Permanent wave	176	(16.7)
Dyeing/Tinting	36	(3.4)
Washing	200	(19.0)
Drying	22	(2.1)
Work tenure (years)^†^		
< 3	121	(14.1)
< 10	336	(39.0)
≧ 10	404	(46.9)
Hours worked per week		
< 52	234	(25.5)
≧ 52	682	(74.5)
Exposure to hairdressing chemicals		
Not exposed	815	(80.2)
Exposed	201	(19.8)
Personal protective equipment used		
No	531	(52.4)
Yes	482	(47.6)
Types of protective equipment used		
Mask	501	(49.6)
Gloves	463	(45.8)
Other^‡^	46	(4.6)

^*^The total of each variable is not always 1,054 due to missing values.

^†^Work tenure in the same profession.

^‡^Arm sleeves, apron, etc.

### General characteristics of study subjects by dermatologic symptoms

Dermatologic symptoms were more prevalent in younger (28.1%), not married (22.3%) or highly educated (23.6%) hairdresser groups. There were no significant differences in the incidence of dermatologic symptoms according to gender, or BMI. Hairdressers with a bad perceived state of health showed a higher incidence of dermatologic symptoms (29.4%), but there were no significant differences in the incidence of dermatologic symptoms according to smoking status, weekly alcohol consumption or the amount of regular exercise (Table 3).

**Table 3:** Demographic and health characteristics of study subjects by dermatologic symptoms

Variable^*^	**Dermatologic symptoms**^**†**^**(+)**		**Dermatologic symptoms (−)**		**p value**^**‡**^
	**n**	**(%)**	**n**	**(%)**	
Age					
< 30	96	(28.1)	246	(71.9)	< 0.001
< 40	67	(20.1)	267	(79.9)	
≧ 40	45	(12.9)	304	(87.1)	
Gender					
Male	32	(21.2)	119	(78.8)	0.741
Female	179	(20.0)	715	(80.0)	
BMI					
< 25	181	(19.8)	735	(80.2)	0.622
≧ 25	7	(16.7)	35	(83.3)	
Marital status					
Not married^§^	132	(22.3)	459	(77.7)	0.015
Married	66	(16.1)	344	(83.9)	
Educational level					
≦ High school completion	101	(17.8)	465	(82.2)	0.023
>High school completion	111	(23.6)	360	(76.4)	
Smoking status					
Non/past smoker	157	(19.5)	648	(80.5)	0.163
Current smoker	51	(23.8)	163	(76.2)	
Weekly alcohol consumption^||^					
None	88	(19.4)	365	(80.6)	0.408
Mild	97	(21.1)	363	(78.9)	
Heavy	20	(26.0)	57	(74.0)	
Weekly exercise					
None	140	(22.6)	479	(77.4)	0.072
≦ 4times	58	(16.8)	287	(83.2)	
> 4times	9	(16.1)	47	(83.9)	
Perceived state of health					
Good	62	(15.1)	348	(84.9)	0.001
Normal	106	(21.7)	383	(78.3)	
Bad	40	(29.4)	96	(70.6)	

^*^The total of each variable is not always 1,054 due to missing values.

^†^Characteristic dermatologic symptoms (itching, redness, swelling, cracking, blister formations) lasting for longer than 3 weeks.

^‡^P-value by chi-squared test, p < 0.05.

^§^Includes divorced, separated and bereaved.

^||^None: never drink or only on rare occasions, Mild: < 4 times per week, Heavy: ≧ 5 times.

### Work characteristics of study subjects by dermatologic symptoms

Dermatologic symptoms were more prevalent in hairdressers who work in franchise hair salons (22.6%). Staff hairdressers and those whose work tenure was less than 3 years also showed a higher prevalence of dermatologic symptoms (25.6%). Regarding the main work tasks, dyeing/tinting (30.6%) and washing (32.5%) were associated with a higher incidence of dermatologic symptoms than cutting (16.9%) or giving a permanent wave (16.5%). Other than these variables, there were no significant differences in the incidence of dermatologic symptoms according to working hours, exposure to hairdressing chemicals, whether personal protective equipment was used, or the type of personal protective equipment used (Table 4).

**Table 4:** Work characteristics of study subjects by dermatologic symptoms

Variable^*^	**Dermatologic Symptoms**^**†**^**(+)**		**Dermatologic Symptoms (−)**		**p value**^**‡**^
	**n**	**(%)**	**n**	**(%)**	
Business type					
Franchise hair salons	98	(22.6)	335	(77.4)	0.035
Private hair salon	103	(17.3)	491	(82.7)	
Salon size (number of employees)					
1	29	(12.7)	199	(87.3)	0.001
2 - 4	42	(16.9)	206	(83.1)	
5 - 9	33	(22.6)	113	(77.4)	
≧ 10	88	(25.4)	259	(74.6)	
Training status					
Master	52	(13.4)	336	(86.6)	< 0.001
Designer	76	(20.0)	304	(80.0)	
Staff	84	(29.4)	202	(70.6)	
Main task					
Cutting	105	(16.9)	515	(83.1)	< 0.001
Permanent wave	29	(16.5)	147	(83.5)	
Dyeing/Tinting	11	(30.6)	25	(69.4)	
Washing	65	(32.5)	135	(67.5)	
Drying	2	(9.1)	20	(90.9)	
Work tenure (years)^§||^					
< 3	31	(25.6)	90	(74.4)	0.011
< 10	69	(20.5)	267	(79.5)	
≧ 10	59	(14.6)	345	(85.4)	
Hours worked per week					
> 52	44	(18.8)	190	(81.2)	0.508
≧ 52	142	(20.8)	540	(79.2)	
Exposure to hairdressing chemicals					
Not exposed	172	(21.1)	643	(78.9)	0.315
Exposed	36	(17.9)	165	(82.1)	
Personal protective equipment used					
No	104	(19.6)	427	(80.4)	0.588
Yes	101	(21.0)	381	(79.1)	
Types of protective equipment used					
Mask	108	(21.6)	393	(78.4)	0.460
Gloves	89	(19.2)	374	(80.8)	
Other ^||^	7	(15.2)	39	(84.8)	

^*^The total of each variable is not always 1,054 due to missing values.

^†^Characteristic dermatologic symptoms (itching, redness, swelling, cracking, blister formations) lasting for longer than 3 weeks.

^‡^P-value by chi-squared test, p < 0.05.

^§^Work tenure in the same profession.

^||^Arm sleeves, apron, etc.

### Distribution of characteristics of dermatologic symptoms of the study subjects according to training status

Dermatologic symptoms were more prevalent in the staff hairdressers (39.6%), and the most commonly affected body parts were forearms or fingers (71.0%), regardless of the training status. In response to the questions regarding the specific types of dermatologic symptoms experienced, designer hairdressers reported a statistically higher prevalence of symptoms, except for blister formation, while staff hairdressers showed the highest prevalence of symptoms lasting for more than 3 weeks (39.2%). The proportion of hairdressers who had needed a hospital visit due to dermatologic symptoms in the past year was significantly higher in master hairdressers (47.8%), and dermatologic disease diagnosed by physicians (47.1%), or dermatologic symptoms that were relieved on the weekend (when hairdressers were not working) (46.6%), were also more common in master hairdressers (Table 5).

**Table 5:** Distribution of characteristics of dermatologic symptoms of the study subjects according to training status

Variable^*^	**Masters**		**Designers**		**Staff**		**p value**^**†**^
	**n**	**(%)**	**n**	**(%)**	**n**	**(%)**	
Dermatologic symptoms^‡^							
No	336	(39.9)	304	(36.1)	202	(24.0)	< 0.001
Yes	52	(24.5)	76	(35.9)	84	(39.6)	
Affected body part							
Whole body	11	(40.7)	12	(44.4)	4	(14.8)	0.016
Face, neck	23	(35.9)	21	(32.8)	20	(31.3)	
Forearms, fingers	99	(28.5)	127	(36.5)	122	(35.1)	
Trunk, shoulders	1	(10.0)	5	(50.0)	4	(40.0)	
Thighs, legs	9	(47.4)	5	(26.3)	5	(26.3)	
Feet, toes	14	(63.6)	6	(27.3)	2	(9.1)	
Specific symptoms							
Redness and swelling							
No	242	(35.6)	262	(38.6)	175	(25.8)	0.002
Yes	69	(25.0)	111	(40.2)	96	(34.8)	
Redness and cracking							
No	251	(32.9)	302	(39.6)	210	(27.5)	0.039
Yes	47	(26.1)	67	(37.2)	66	(36.7)	
Blister formation							
No	245	(31.7)	310	(40.2)	217	(28.1)	0.556
Yes	56	(32.2)	63	(36.2)	55	(31.6)	
Redness and itching							
No	225	(36.8)	236	(38.6)	151	(24.7)	0.001
Yes	101	(27.3)	140	(37.8)	129	(34.9)	
Symptoms lasting > 3 weeks							
No	179	(43.3)	140	(33.9)	94	(22.8)	< 0.001
Yes	56	(25.8)	76	(35.0)	85	(39.2)	
Hospital visit^§^							
No	57	(23.7)	103	(42.7)	81	(33.6)	< 0.001
Yes	160	(47.8)	88	(26.3)	87	(26.0)	
Diagnosed by physician^||^							
No	49	(19.7)	117	(47.0)	83	(33.3)	< 0.001
Yes	128	(47.1)	80	(29.4)	64	(43.5)	
Symptoms abated^¶^							
No	87	(27.7)	118	(37.6)	109	(34.7)	< 0.001
Yes	109	(46.6)	68	(29.1)	57	(24.4)	

^*^The total of each variable is not always 1,054 due to missing values.

^†^P-value by chi-squared test, p < 0.05.

^‡^Characteristic dermatologic symptoms (itching, redness, swelling, cracking, blister formations) lasting for longer than 3weeks.

^§^Requirement for hospital visit in the past year due to dermatologic symptoms.

^||^Dermatitis or eczema diagnosed by a physician (s) while working as a hairdresser.

^¶^Dermatologic symptoms abated over the weekends or when off-duty.

### Odds ratios of dermatologic symptoms according to exposure to hairdressing chemicals, training status and main tasks

Table 6 shows the odds ratios of dermatologic symptoms according to exposure to hairdressing chemicals, training status and main tasks in three different models. Regarding exposure to hairdressing chemicals, no statistically significant differences were observed. Regarding training status, master hairdressers were set as a reference group, and regarding main tasks, hairdressers who performed cutting work were set as a reference group.

**Table 6:** Odds ratios of dermatologic symptoms according to exposure to hairdressing chemicals, training status and main tasks (n = 1,054)

Variable*	**Crude**	**95% CI**	**Model I**	**95% CI**^**†**^	**Model II**	**95% CI**^**‡**^
Exposure to chemicals						
No	1.00		1.00		1.00	
Yes	0.82	0.55 - 1.21	0.91	0.56 - 1.47	0.89	0.53 - 1.49
Training status						
Master	1.00		1.00		1.00	
Designer	1.62	1.10 - 2.38	1.31	0.77 - 2.22	1.22	0.68 - 2.19
Staff	2.69	1.82 - 3.96	2.83	1.47 - 5.43	2.70	1.32 - 5.51
Main task						
Cutting	1.00		1.00		1.00	
Permanent wave	0.97	0.62 - 1.52	0.93	0.57 - 1.51	0.89	0.53 - 1.49
Dyeing/tinting	2.16	1.03 - 4.52	1.00	0.36 - 2.81	1.08	0.38 - 3.07
Washing	2.36	1.64 - 3.39	2.14	1.32 - 3.47	2.03	1.22 - 3.37
Drying	0.49	0.11 - 2.13	0.45	0.10 - 2.02	0.45	0.10 - 2.03

^*^The total of each variable is not always 1,054 due to missing values.

^†^Odds ratio and 95% confidence intervals estimated using logistic regression model adjusted for age, gender, marital status, educational level, smoking status, weekly alcohol consumption, amount of regular exercise per week, perceived state of health.

^‡^Odds ratio and 95% confidence intervals estimated using logistic regression model adjusted for age, gender, marital status, educational level, smoking status, weekly alcohol consumption, amount of regular exercise per week, perceived state of health, business type, exposure to hairdressing chemicals, personal protective equipment.

According to the results of crude analysis for training status, higher odds ratios were observed in designer hairdressers (1.62, 95% CI: 1.10 - 2.38) and staff hairdressers (2.69, 95% CI: 1.82 - 3.96), respectively. In Model I, higher odds ratios were observed in both designer hairdressers (1.31, 95% CI: 0.77 - 2.22) and staff hairdressers (2.83, 95% CI: 1.47 - 2.43), and in Model II, higher odds ratios were also observed in both designer hairdressers (1.22, 95% CI: 0.68 - 2.19) and staff hairdressers (2.70, 95% CI: 1.32 - 5.51), although the results were statistically significant only in staff hairdressers.

Regarding the main tasks, compared to the cutting group, the dyeing/tinting group (2.16, 95% CI: 1.03 - 4.52) and the washing group (2.36, 95% CI: 1.64 - 3.39) showed higher odds ratios in the crude model. In the case of Model I, only the washing group (2.14, 95% CI: 1.32 - 3.47) showed higher odds ratios, and in the case of Model II, the dyeing/tinting group (1.08, 95% CI: 0.38 - 3.07) and the washing group (2.03, 95% CI: 1.22 - 3.37) showed higher odds ratios, although the results were statistically significant only in the washing group (Table 6).

## Discussion

The relationship between work characteristics such as training status and main task and dermatologic symptoms in hairdressers in Korea were evaluated in this study by analyzing the results of a self-reported questionnaire-based survey conducted in 2012. In this study, 20.1% (212 hairdressers) of the study subjects were categorized into a dermatologic symptom-positive group, showing consistency with the results of previous studies that show 10% to 20% symptom prevalence [20,23], although some studies concerning only acute dermatologic symptoms reported a higher symptom prevalence. Regarding training status, while 13.4% of master hairdressers were categorized into the symptom-positive group, 29.4% of staff hairdressers fell into this group (P-value < 0.001).

Several previous studies have suggested a positive association between wet work and dermatologic symptoms in hairdressers; however, work characteristics which could have an effect on dermatologic symptoms could vary according to the social atmosphere and this may lead to previous results that contrast with those of our study [24,25]. Compared to other studies dealing with health issues of hairdressers, which usually involve several hundreds of participants, a relatively large number of study subjects was used for analysis in the present study. In addition, the present study is the first to confirm the relationship between the training status and main tasks of hairdressers and to concurrently assess the relationship between work characteristics and dermatologic symptoms of hairdressers in Korea.

The main tasks performed in hair salons in Korea can be grouped into 4 categories: cutting, giving a permanent wave, dyeing/tinting, and washing/drying. Cutting work involves repetitive manual movement with arms in a raised position and the use of equipment such as scissors or razors, which increases vulnerability to musculoskeletal disease or occupational injuries [7]. Permanent waves or dyeing/tinting involve the use of various kinds of chemicals. Exposure to hairdressing chemicals such as dyeing or tinting agents is likely to induce an acute form of dermatologic symptoms [26,27]. On the other hand, relatively weak chemicals, such as detergents used in washing work, are more likely to act as a chronic form of irritant and induce a chronic form of dermatologic symptoms. Additionally, wet work acts as a weak but chronic irritant which can perturb the skin barrier [13], and it plays a prominent role in inducing dermatologic symptoms [15].

In considering whether hairdressers are actually exposed to an extended duration of wet work, one report suggested that hairdressers are exposed to more than 2 hours of wet work per day and that exposure time was longer in master hairdressers than in apprentices [5], while another report suggested that wet work duration is related not only to washing but also to water-resistant glove wearing tasks such as dyeing or giving permanent waves [24]. Likewise, as a result of prolonged exposure to chemicals and water, the occupational group with the highest annual incidence rate (120 cases per 100,000 employed) of occupational contact dermatitis was reported to be female hairdressers and barbers [28]. Despite the consistent reports of a high prevalence of dermatologic symptoms in hairdressers, the results of the studies of the work characteristics that could have an effect on the incidence of dermatologic symptoms vary.

In the present study, the relationships between the main tasks and the training status of hairdressers were evaluated. The results of this analysis showed that the main tasks, which are known to involve a significant amount of wet work, were determined according to the training status. Thus, training status could potentially represent the duration of wet work in hairdressers. In detail, the results of this study showed that 51.9% and 43.4% of master and designer hairdressers, respectively, reported cutting work to be their main task, while only 4.7% of staff hairdressers reported cutting work to be their main task. Regarding main tasks such as giving a permanent wave or dyeing/tinting, which likely involve exposure to hairdressing chemicals, 46.7% of designer hairdressers reported these to be their main tasks, while 28.8% of master and 24.5% of staff hairdressers reported these to be their main tasks. However, regarding washing, which is believed to be the main source of wet work [24], while only 1.5% and 2.0% of master and designer hairdressers reported this to be their main task, 96.5% of staff hairdressers reported this to be their main task, thus indicating an obvious difference in main tasks according to the training status. These findings could result from the circumstances such that the majority of staff hairdressers belong to large-sized franchise hair salons in which the division of duties is clear. Since there was a significant difference in main tasks according to the training status, training status was used to represent the potential duration of wet work. Additionally, since the results of previous studies suggest that glove-requiring tasks such as dyeing or permanent waves are also a potential source of wet work [24], the burden of wet work of staff hairdressers would be even greater than expected. Regarding the incidence of the diagnosis of dermatitis by a physician and of a hospital visit due to dermatitis while working as a hairdresser, higher rates were shown in the master hairdresser group. These two questionnaire items represent the utilization of medical services or accessibility to medical services rather than the seriousness of dermatologic symptoms, since accessibility to medical service are low in staff hairdressers due to lack of autonomy at work. The seriousness of any dermatologic symptoms could be inferred from the responses to the questions regarding specific dermatologic symptoms and the incidence of symptoms lasting for more than 3 weeks. As the results in Table 3 show, all the dermatologic symptoms except blister formations were more prevalent in staff than master hairdressers.

There are potential limitations in this study that should be considered. First, the study was conducted based on a self-reported questionnaire that targeted the experience in the past year of dermatologic symptoms, which was thus bound to the possibility of recall bias. Second, questions about participants’ past or family medical history, including the incidence of dermatitis or atopic disease, which could be related to the current condition of any dermatologic symptoms, were not included in the questionnaire. Third, information on the general characteristics of individuals who did not respond to the questionnaire was absent. The survey was conducted during a 5-month period, and with the aid of relevant hairdresser associations and academic organizations, a relatively high response rate of 80.2% could be achieved; however, a certain degree of selection bias could still remain and not be controlled for. Furthermore, as in other cross-sectional studies, this study is not free from the healthy worker effect [29]. There are reports suggesting that hand eczema is one of the recognizable reasons for a change of job in hairdressers [25]. On the contrary, other studies suggest that mild forms of dermatitis alone do not contribute significantly to a job change in hairdressers [24,30], but that but more serious health problems such as asthma or chronic obstructive pulmonary disease (COPD) act as important factors in the changing of a job [31]. The results of previous studies suggest that, although dermatitis contributed to around 30% of career changes in student hairdressers [24], other work characteristics seemed to be playing a greater role in changing of a job in hairdressers. At the same time, due to the strict hierarchical structure of the work environment for hairdressers, the selective migration of the affected workers from a risk factor-exposed task (e.g. washing) to a non-exposed task (e.g. cutting) is unlikely to happen. Finally, a causal relationship or dose–response relationship between training status or main task and dermatologic symptoms could not be clearly identified due to the cross-sectional study design and a lack of quantitative measures to evaluate actual wet work durations according to work characteristics. Since self-employed master hairdressers are supposed to perform every step of the hairdressing, this group was considered as a potential high risk group at the beginning of the study, but the results of subgroup analysis did not show sufficient statistical evidence to suggest an elevated risk for dermatologic symptoms in self-employed master hairdressers. Differences in workload due to salon size or autonomy at work might have contributed to this result.

In nations such as the United Kingdom or Germany, guidelines and management plans for dermatologic health of hairdressers exist [32]. According to the report on the nationwide industry survey by Statistics Korea, although the need for the hairdressing industry is continually increasing in Korea [7], no specific guidelines or health management plans exist to promote the dermatologic health of hairdressers.

Hairdressers experience numerous types of health problems and are exposed to a wide variety of chemical, physical, and psycho-sociological hazards. Among these health issues, dermatologic symptoms account for a significant proportion, thus the potential impact of the results of this study should be considered carefully. Unfortunately, there are no suitable guidelines or manuals available to promote the dermatologic health of hairdressers in Korea except the Industrial Accident Prevention Guidelines for hairdressers. This study could provide a basis for the establishment of dermatologic health management plans for hairdressers.

## Conclusions

This study suggested that the training status of Korean hairdressers is closely linked to their main tasks, and that these two work characteristics are closely related to the incidence of dermatologic symptoms, even after adjustment for confounding variables. According to the results of our study, wet work seems to be strictly reserved for hairdressers who hold a certain training status, and acts as a greater risk factor in the dermatologic health of hairdressers in Korea. In the future, based on the results of this study, research that involves quantitative measures to evaluate actual wet work duration according to work characteristics such as training status will be required, and health management guidelines for hairdressers should be established.

## Competing interest

The authors declare that they have no competing interest.

## Authors’ contributions

PKJ: The first author of this article. He designed this research, collected and interpreted the data, suggested the causal relationship, prepared the draft of this manuscript, and approved the final version of the manuscript. JHL: He revised the draft of this manuscript, and approved the final version of the manuscript. JHB: She collected the data and prepared the draft of the manuscript, and approved the final version of the manuscript. JH: He analyzed the tasks of hairdressers and working environment, revised the draft of this manuscript, and approved the final version of the manuscript. JUW: He advised the causal relationship, revised the draft of this manuscript, and approved the final version of the manuscript. IK: She suggested the design of this research, advised the causal relationship, interpreted data, revised the draft of this manuscript, and approved the final version of the manuscript. JR: The corresponding author of this article. He suggested the design of this research, interpreted data, revised the draft of this manuscript, and approved the final version of the manuscript.
